# MicroRNA profiling of *tomato leaf curl new delhi virus *(tolcndv) infected tomato leaves indicates that deregulation of mir159/319 and mir172 might be linked with leaf curl disease

**DOI:** 10.1186/1743-422X-7-281

**Published:** 2010-10-25

**Authors:** Afsar R Naqvi, Qazi MR Haq, Sunil K Mukherjee

**Affiliations:** 1 Plant Molecular Biology Group, International Centre for Genetic Engineering and Biotechnology, Aruna Asaf Ali Marg, New Delhi-110067, India; 2 Department of Biosciences, Jamia Millia Islamia (A Central University), New Delhi- 110025, India

## Abstract

**Background:**

Tomato leaf curl virus (ToLCV), a constituent of the genus *Begomovirus*, infects tomato and other plants with a hallmark disease symptom of upward leaf curling. Since microRNAs (miRs) are known to control plants developmental processes, we evaluated the roles of miRNAs in *Tomato leaf curl New Delhi virus *(ToLCNDV) induced leaf curling.

**Results:**

Microarray analyses of miRNAs, isolated from the leaves of both healthy and ToLCNDV agroinfected tomato cv Pusa Ruby, revealed that ToLCNDV infection significantly deregulated various miRNAs representing ~13 different conserved families (e.g., miR319, miR172, etc.). The precursors of these miRNAs showed similar deregulated patterns, indicating that the transcription regulation of respective miRNA genes was perhaps the cause of deregulation. The expression levels of the miRNA-targeted genes were antagonistic with respect to the amount of corresponding miRNA. Such deregulation was tissue-specific in nature as no analogous misexpression was found in flowers. The accumulation of miR159/319 and miR172 was observed to increase with the days post inoculation (dpi) of ToLCNDV agroinfection in tomato cv Pusa Ruby. Similarly, these miRs were also induced in ToLCNDV agroinfected tomato cv JK Asha and chilli plants, both exhibiting leaf curl symptoms. Our results indicate that miR159/319 and miR172 might be associated with leaf curl symptoms. This report raises the possibility of using miRNA(s) as potential signature molecules for ToLCNDV infection.

**Conclusions:**

The expression of several host miRNAs is affected in response to viral infection. The levels of the corresponding pre-miRs and the predicted targets were also deregulated. This change in miRNA expression levels was specific to leaf tissues and observed to be associated with disease progression. Thus, certain host miRs are likely indicator of viral infection and could be potentially employed to develop viral resistance strategies.

## Background

Geminiviruses are plant pathogens that profoundly affect diverse plant crops in tropical and subtropical countries [1-3]. These are emerging class of viruses with new strains still evolving, thereby making them more virulent with wide host range specificity [4,5]. *Tomato leaf curl New Delhi virus *(ToLCNDV) is a member of *begomovirus *genus infecting tomato crop and it causes severe yield loss. This group of viruses may have monopartite (DNA A) or bipartite (DNA A and DNA B) circular ssDNA genomes. The DNA A component encodes six Open Reading Frames (ORFs) namely AC1, AC2, AC3, AC4, AV1 and AV2 while only two proteins (BC1 and BV1) are encoded by DNA B. These ORFs are encoded either in the virion or complementary-sense orientations. Most of these proteins have been implicated in virus multiplication and pathogenesis. One of the apparent symptoms associated with ToLCNDV infection is upward leaf curling in tomato leaves.

MicroRNAs (miRNAs) have recently emerged as the key regulatory molecules in diverse biologically relevant processes, both in plants and animals [6,7]. The miRNAs are transcribed from their own promoters by RNA pol II activity and have characteristic 5' cap and 3' poly-A tail [8,9]. These pri-miRNA transcripts form hairpin like structure and are sequentially processed by the action of RNase III-like proteins, namely HYL1/SER1 and DCL1 in *Arabidopsis*, to generate miRNA duplexes [6,10]. The mature miRNA enters into a multi-protein complex termed RNA-induced silencing complex (mi-RISC) and guides it to the target mRNAs with complementary sequences. This consequently leads to the target cleavage [8,11] and/or inhibits translation of the targets [12]. In plants, miRNAs have been demonstrated to participate in leaf morphogenesis, phase transition, flower development and root and shoot development [13-18]. It is thus apparent that ToLCNDV induced leaf curling in tomato can be utilized as a model system to study the influence of miRNA-mediated biological actions on leaf deformations.

In *Arabidopsis*, few miRs have been demonstrated to critically regulate leaf development *viz*., miR165/166, miR164 and miR319/159 [19-21]. For instance, miR165/166 targeted HD-ZIP III transcription factors (TFs) are involved in determining adaxial and abaxial pattern formation [20] while, miR159 and miR319 play important roles in maintaining leaf phenotype by regulating members of MYB transcription factors and TCP transcription factors, respectively [19]. Similarly, miR164 that targets CUC2 also takes care of leaf patterning by controlling serration of leaf margins [21]. The involvement of these miRNAs in leaf morphology has been demonstrated by raising *Arabidopsis *transgenic over-expressing miRNAs or targets with mutated miRNA binding sites and these transgenic plants revealed clear leaf development associated defects. Moreover, evidences support the involvement of miRNAs in biotic and abiotic stresses. For instance, miR393 expression is induced under bacterial infection [22]. The F-box auxin receptor proteins targeted by miR393 are consequently down-regulated, thereby suppressing auxin signaling pathways and probably conferring resistance against pathogens. On the other hand, miR395, miR399, miR398, etc., have been associated with specific abiotic stresses [7,23,24].

Viral encoded proteins interfere with host RNAi pathways and thus these proteins distort the normal cellular activities [25-27]. The Viral Suppressors of RNA silencing (VSRs) are crucial in disease severity and developmental abnormalities [25]. ToLCNDV encodes three VSRs *viz*., AC2, AC4 and the pre-coat protein AV2 [26,27]. AC2 is a crucial VSR that disturbs post-transcriptional gene silencing and mutation in AC2 leads to reduced pathogenesis [28], Karjee et al. Unpublished data]. Further, over-expression of AC2 induces expression of several host genes [29] and impacts severely on plant architecture. *African cassava mosaic virus *(ACMV) AC4, on the other hand, has been shown to bind directly to miRNAs, thereby making mi-RISC non-functional [27].

ToLCNDV is also accompanied by a satellite DNA (β DNA) that encodes βC1 protein [30]. Recently, it has been demonstrated that βC1 of *Tomato yellow leaf curl China New virus *(TYLCCNV) interacts with host ASYMMETRIC LEAVES 1 (AS1) to alter leaf phenotype [31]. All these studies suggest that ToLCNDV encodes enough proteins to help virus achieve suitable environment for its survival and cause pathogenesis. The VSRs influence miRNA biogenesis of host, leading to developmental abnormalities [32]. Although viral proteins, VSRs, have been shown to hamper the development of transgenic plants [Karjee et al. Unpublished data], not much has been studied about which miRNAs are responsible for ToLCNDV induced changes in leaf phenotype. We attempted to understand how ToLCNDV utilizes host miRNAs to bring about curl phenotype in the leaves. Since the involvement of miRNA in biotic responses and leaf patterning is now well recognized, we wanted to explore the molecular principles behind the ToLCNDV mediated leaf abnormality.

Here, we report that ToLCNDV agroinfection can significantly deregulate the host miRNA expression and the corresponding targets as well. Moreover, this ToLCNDV induced differential shift in miRNA levels was specific to leaf tissues since we did not observe the similar changes of either miRNA or its target in tissues other than the leaves. This tissue specific deregulation of miRNA levels is perhaps required to establish favorable conditions for virus survival and perhaps leads to leaf deformation. The expression levels of miR159/319 and miR172 were observed to be associated with disease progression, thereby making these as potential biomarkers for ToLCNDV infection.

## Results

### PCR- and Rolling Circle Amplification (RCA) - based detection of virus in tomato tissues

**Figure 1:**
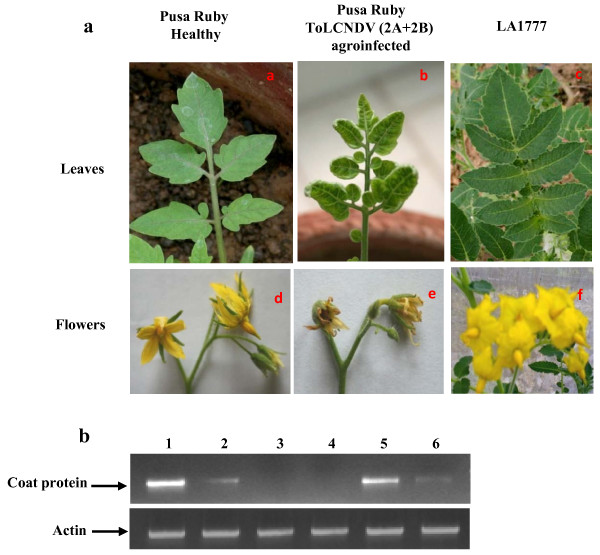
**Phenotype of the tomato plants under study and the PCR based detection of viral DNA fragment in different tissues of ToLCNDV (2A+2B) agroinfected Pusa Ruby**. **a**, Photographs of different leaves and flowers of tomato plant *viz*., Healthy tomato (cv Pusa Ruby) leaves (a) and flowers (d); ToLCNDV (2A+2B) infected tomato (cv Pusa Ruby) leaves exhibiting upward curling (b) and flowers (e); and Tomato accession LA1777 leaves (c) and flowers (f). **b**, Agarose gel showing amplification of Coat Protein (CP) of ToLCNDV using gDNA isolated from leaves (lane 1), flowers (lane 2), stem (lane 5) and fruits (lane 6) tissues of tomato plants agroinfected with ToLCNDV (2A+2B) while, leaves (lane 3) and flowers (lane 4) of healthy plants were used as negative controls. Amplification of CP in the lanes reflects the presence of virus in the respective tissues. Actin gene amplification served as an internal loading control.

Using the agroinfiltration technique, we were able to induce the expression of leaf curl disease in tomato cv Pusa Ruby. For these experiments, we used two separate constructs containing the dimer of ToLCNDV A and ToLCNDV B genomes that were individually cloned in the pCAMBIA based binary vector. The leaves of ToLCNDV (2A+2B) agroinfiltrated plants exhibited curled and mottled morphology along with the yellowing in the veins (Figure 1a) and these plants showed stunted growth. The accumulation of the viral DNA (~2.5 kb) was detected by carrying out rolling circle amplification (RCA) of the genomic DNA obtained from the agroinfiltrated plants (see Additional file 1; Fig. S 1, lanes 1 and 3); while such amplification was not found in the healthy controls (see Additional file 1; Fig. S 1, lane 2).

The morphology of the leaves and flowers of healthy Pusa Ruby, ToLCNDV (2A+2B) agroinfected Pusa Ruby and LA1777 plant is shown in Figure 1a. The amplification of CP gene (~500 bp) was observed in different tissues samples of ToLCNDV (2A+2B) infected plants. We noticed preferential abundance of the virus in leaf tissues (Figure 1b). As reflected from these data, the virus was abundantly present in leaves and stem (Figure 1b, lanes 1 and 5) but rarely present in flowers and fruit tissues (Figure 1a, lane 2 and 6). Since we were interested in identifying the leaf specific changes in miRNAs after ToLCNDV infection, we chose the flowers as the control tissues.

### Microarray analysis

To determine the changes in the global miRNA expression in ToLCNDV (2A+2B) agroinfected leaves, microarray experiments were carried out with the total RNA isolated from leaves of healthy and ToLCNDV agroinfected tomato cv Pusa Ruby, an elite tomato cultivar showing extreme sensitivity to ToLCNDV. We selected tomato accession LA1777, a variety of non-elite tomato that shows resistance to ToLCNDV as the vector whitefly (*Bemisia tabaci*) can not feed well on it. For comparison, we also used ToLCNDV (2A+2B) agroinfiltrated tomato cv 15 SB SB leaves (7dpi) and the mock agroinfiltrated leaves of the same cultivar.

**Figure 2:**
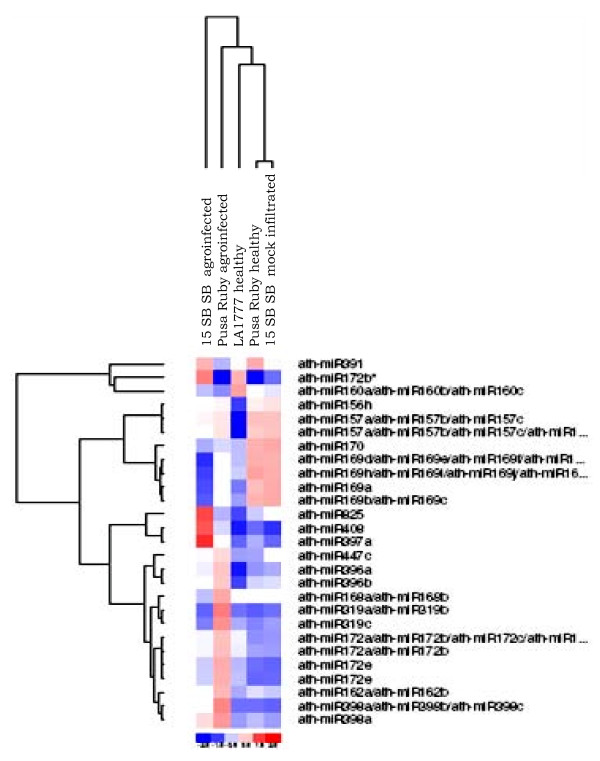
**Heat map obtained from the microarray data showing the differentially expressed miRNAs in ToLCNDV (2A+2B) infected leaves compared to other leaf samples**. Ath-miR stands for microRNA of *Arabidopsis thaliana *plant. The color scale shown at the bottom illustrates the relative expression level of a miRNA across all samples: red represents an expression level above mean, blue represents expression lower than the mean. The clustering is performed on log_2 _(Hy3/Hy5) ratio which passed the filtering criteria on variation across samples; standard deviation > 0.50.

Since only few miRNAs were available in tomato small RNA database, the miRNA profiling was done using probes designed from miRNA sequences for all the plant species available on Sanger's database (Release 12, http://microrna.sanger.ac.uk/). The microarray analysis was performed and data were analyzed at EXIQON, Denmark and the methodology is briefly discussed in the experimental procedures. The heat map of the array clearly suggests that miRNAs belonging to 13 conserved families are differentially expressed under ToLCNDV (2A+2B) infection. Most of the miRNAs listed in the heat map were up-regulated with statistical significance and these include miR162, miR168, miR172, miR319, miR396, miR397, miR398, miR408 and miR447 (Figure 2). Besides, we also observed down-regulation of few miRNAs *viz*., miR160, miR169, miR170 and miR391 following ToLCNDV infection (Figure 2).This observation assumes much significance as the ToLCNDV genome encode at least three VSRs, namely AC2, AC4 and AV2 (or the pre-coat protein). The miRNAs altered under ToLCNDV infection and their respective predicted targets alongwith their putative functions are listed in Table 1. Only those miRNAs are included in the list that are conserved between *Arabidopsis *and tomato and also shows significant changes in miRNA expression pattern in response to ToLCNDV infection.

**Table 1:** List of miRNAs found to be differentially deregulated under ToLCNDV (2A+2B) agroinfection in microarray analysis.

miRNA	Fold (↑ or ↓)	Target protein class	Function	References
miR160	↓, 2X	Auxin response factors	Hormone signaling and plant development	[50]

miR162	↑, 2X	Dicer-like (DCL) protein	Plant development	[39]

miR168	↑, 2X	ARGONAUTE (AGO) protein	Plant development	[40,51]

miR169	↓, 2X	CBF HAP2-like factors	Abiotic stress responses	[52]

miR171	↓, 2X	Scarecrow- like (GRAS domain) TFs	Flowering time	[11]

miR172	↑, ~4X	APETALA-2 (AP2) like TFs	Floral identity and phase transition	[13,17]

miR319	↑, ~4X	TCP, bHLH TF	Leaf patterning	[19]

miR391	↓, 3X	Not known	Not known	

miR396	↑, 2X	GRL TFs, Rhodanase like proteins	Defense responses	[23]

miR397	↑, 1.5X	Laccases, b -6 tubulin	Fungal infection	[7,23]

miR398	↑, 3X	Copper superoxide dismutases (CSD1/2)	Abiotic stress	[43]

miR408	↑, 1.5X	Plantacyanin	Stress responses	[23]

miR447	↑, 2.5X	2-Phosphoglycerate kinase	Metabolic pathway	[7]

The relative expression values of the individual miRNAs as revealed from the array analysis have been plotted as a histogram (see Additional file 1; Fig. S 2 a, b). However, it should be noted that the tomato cv 15 SB SB leaves agroinfiltrated with ToLCNDV (2A+2B) construct did not show similar deregulation in the miRNA expression when compared with ToLCNDV agroinfected Pusa Ruby leaves. This could be due to the fact that either the 15 SB SB plants could not be efficiently influenced by agroinfiltration of ToLCNDV genome or perhaps this tomato variety behaved differently compared to the Pusa Ruby cultivar. Of these two possibilities, the latter one seems to be stronger as the miRNA expression profiles of these two varieties were different even in the non-infected conditions (Figure 2).

### Northern hybridization analysis of microRNAs

To validate the results obtained from microarray analysis and to test the conservation of miRNAs between *Arabidopsis *and tomato, we performed northern hybridization experiments and checked the expression of representative miRNAs in healthy as well as ToLCNDV (2A+2B) agroinfected tomato leaves. We used miR159, miR164, miR170/171, miR172 and miR319 for these studies as they are involved in leaf/shoot development as well as stress responses [19-21]. It may be noted that miR159 and miR171 show high sequence similarity to miR319 and miR170, respectively. The microarray data revealed that both miR319 and miR170 were significantly altered during ToLCNDV agroinfection (Figure 2). Since these miRs have been shown to regulate leaf development, we analyzed their levels by northern analysis.

**Figure 3:**
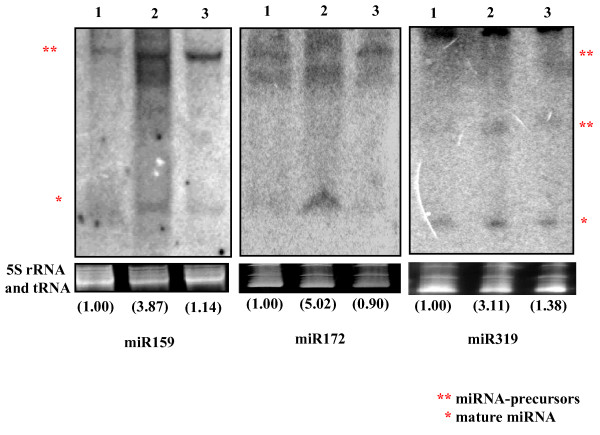
**Northern hybridization analysis of various deregulated miRNAs**. The levels of miR159, miR172 and miR319 were induced following ToLCNDV agroinfection The experiments were conducted thrice. The normalization of individual mature miRNAs was performed with respect to EtBr stained gels, further quantified and averaged. The value corresponding to healthy sample is taken as 1 while the values presented for other samples are shown in parenthesis. EtBr stained gels serve as loading control. The bands highlighted in the blot are marked by asterisks where mature miRNA is represented by (*) while pre-miRNAs are marked by (**). **1**: Pusa Ruby healthy; **2**: ToLCNDV (2A+2B) infected Pusa Ruby; **3**: LA1777 healthy.

As observed in Figure 3, miR159, miR172 and miR319 were up-regulated approximately by 4, 5 and 3 folds, respectively, in ToLCNDV (2A+2B) infected leaves as compared to both healthy leaves and LA1777 leaves (Figure 3, lanes 1 and 3). Such levels of upregulation could be sufficient to affect the corresponding target transcripts levels and thus could be a decisive factor in altering leaf phenotype. MiR164 that regulates leaf pattern through its target CUC2 TFs, showed an almost opposite pattern and was down-regulated by ~2.5 folds in leaves following the ToLCNDV infection (see Additional file, Fig. S 3, lanes 1 and 2). Interestingly, miR164 was almost undetectable in healthy LA1777 leaves (see Additional file, Fig. S 3, middle panel, lane 3) almost mimicking the infected condition of Pusa ruby. Such low expression levels of miR164 in LA1777 could be attributed to its different genetic background. Incidentally, its leaf phenotype is strikingly different from the Pusa Ruby cultivar, as evident from Figure 1a. Similar to miR164, modest reduction (~ 3 folds) was also observed in the expression of miR171 in ToLCNDV infected leaves (see Additional file 1; Fig. S 3, lane 2) compared to both healthy Pusa Ruby and LA1777 leaves. Scarecrow-like TF SCL6 is targeted by miR171 and is demonstrated to play roles in developmental patterning [11]. However, a clear role of these genes in leaf phenotype is not known.

**Figure 4:**
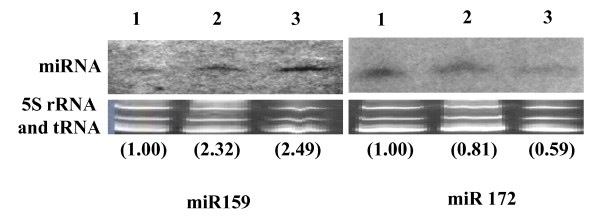
**Northern analysis of miR159 and miR172 levels in flower samples**. Tissues obtained from healthy tomato (cv Pusa Ruby; lane 3), ToLCNDV infected tomato (cv Pusa Ruby; lane 2) and LA1777 (lane 1) plants were checked for the expression of miR159 (1^st ^panel) and miR172 (2^nd ^panel). The northern blot analyses were carried out in two independent sets. For quantification, the expression of miRNA in LA1777 flower samples was taken as standard. The Ethidium bromide stained gels are shown as loading controls.

When flowers of the corresponding plants were analyzed for the expression levels of miR159 and miR172, we observed patterns dissimilar from those of the leaves. The amounts of these two miRs in Pusa Ruby flowers did not show significant changes following ToLCNDV disease expression (Figure 4; lanes 2 and 3). This suggests that ToLCNDV induced deregulation of few mature miRNAs, namely miR159 and miR172, occurred specifically to the leaf tissues.

The interesting observation that came from the northern analysis of miRNAs was that - not only the mature miRs but their corresponding precursor levels were also proportionately altered (Figure 3; notice the bands corresponding to miR-precursors). This indicates that, upon ToLCNDV (2A+2B) agroinfection, the mature miRNA accumulation might be directly related to enhanced formation of pre-miRNAs and *vice versa*. Besides the above-mentioned miRNAs, the levels of few other miRNAs were also checked by northern blot analysis but the data are not presented here as these almost parallel the microarray data. Overall, the northern hybridization analysis of various miRNAs corroborates with the microarray based expression profiling, validating the microarray data.

### Semi-quantitative reverse transcription-PCR of miRNA precursors

**Figure 5:**
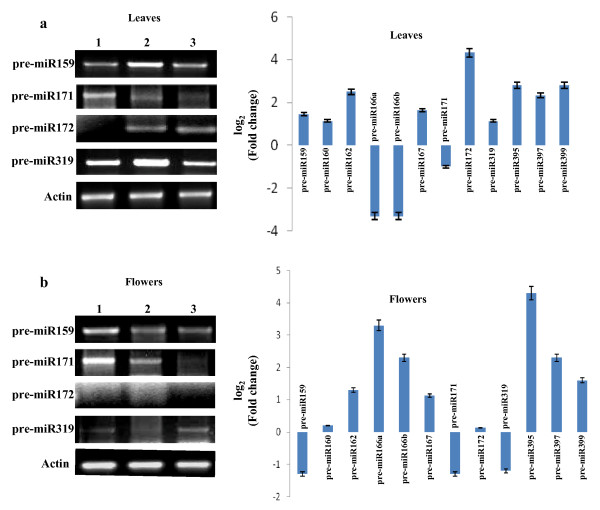
**Semi-quantitative reverse transcription Polymerase chain reaction (RT-PCR) of various pre-miRNAs in (A) leaves and (B) flower tissues of different plants *viz*., Healthy Pusa Ruby (lane 1), ToLCNDV (2A+2B) agroinfected Pusa Ruby (lane 2) and LA1777 (lane 3)**. Upper panel shows pre-miR expression in leaf tissues and the lower panel corresponds to the flower tissues. The band intensity was normalized with respect to actin amplification. The fold change were calculated for ToLCNDV infected samples and is defined as ratio of normalized band intensity for ToLCNDV agroinfected sample (2) to that of healthy sample (1). The histograms were plotted as log_2 _(fold changes) for all the pre-miRNAs studied. The experiments were performed twice with significant reproducibility.

To investigate whether the deregulation of miRNA levels parallel the accumulation level of the pre-miRs, we performed semi-quantitative reverse transcription-PCR of miRNA precursors (pre-miRs). The results of such experiments would hint at the possible processing defect from the pre-miRNA to the mature miRNAs during ToLCNDV disease expression. The pre-miRNA sequences, fetched from miRBase (http://www.mirbase.org/ Release 13), were used to design primers (see Additional file 2; Table S 1) for the amplification of the cDNA, which were prepared from the leaves as well as flowers of healthy Pusa Ruby, ToLCNDV (2A+2B) agroinfected Pusa Ruby and LA1777 tomato plants. Besides the ones that were highlighted in our microarray results, we also included few other pre-miRs *viz*., pre-miR166a, pre-miR166b, pre-miR167 and pre-miR395 (Table 2) in this study since the corresponding mature miRNAs are important in leaf development (miR166) and stress responses (miR167 and miR395). Our data reveal that the expression of most of the pre-miRs was analogous to that of the respective mature miRNA (Figure 5a), indicating that the deregulation perhaps originated in the synthesis of the pre-miRNAs (Compare Table 1 and Table 3, 2^nd ^column) and not in the downstream processing. However, this is not true for all the pre-miRNAs studied. For instance, the microarray data revealed that miR160 expression level in leaves appeared to be reduced by ~1.5 folds following ToLCNDV agroinfection, but the corresponding pre-miRNA levels increased by two folds (Figure 5a). In such cases, ToLCNDV infection perhaps interfered with the processing of mature miRNAs.

**Table 2:** List of miRNAs studied by either northern hybridization or RT-PCR but were not highlighted in the heat map.

miRNA	Target	Function	References
miR166	HD-ZIP TFs	Leaf development	[20]

miR395	ATP Sulfurylase; Sulfate metabolism	Environmental stress response	[7,23]

miR167	ARF TFs	Plant development and hormone signaling	[50]

miR164	NAC domain TFs; CUC1 and CUC2	Leaf development and hormone signaling	[18,21]

miR399	Ubiquitin conjugating enzyme	Phosphate metabolism	[34]

**Table 3:** The table shows the quantitative changes in the expression levels of pre-miRs and miRNA-targeted transcripts in ToLCNDV agroinfected leaves and flowers compared to corresponding Pusa Ruby healthy tissues.

Expression analysis of pre-miR			Expression analysis of miRNA targeted genes		
**Pre-miRNA**	**Leaf (fold ↑/↓)**	**Flower (fold ↑/↓)**	**miRNA targets**	**Leaf (fold ↑/↓)**	**Flower (fold ↑/↓)**

miR159	2.8 ↑	2 ↓	MYB33 like TF	10 ↓	Not studied

miR160	2 ↑	No change	CBF TFs	1.2 ↑	No change

miR162	5 ↑	1.3 ↑	DCL1- like	5 ↓	No change

miR166a	10 ↓	10 ↑	AGO-1 like	2 ↓	Not studied

miR166b	10 ↓	5 ↑	NAM- like TFs	2 ↑	3 ↓

miR167	3 ↑	2 ↑	Squamosa Binding Protein- like TFs	2.5 ↑	Not studied

miR171	2 ↓	2.5 ↓	SCL6- like TFs	2 ↑	10 ↓

miR172	Highly induced	No change	Lanceolate	2.5↓	1.75↑

miR319	2.5 ↑	2 ↓	Apetala 2	4 ↓	No change

miR395	7 ↑	10 ↑	Glucanase	2.5 ↑	No change

miR397	5 ↑	10 ↑	Ubiquitin activating enzyme	2 ↓	1.5 ↓

miR398	5 ↑	10 ↑	CSD1	2 ↓	No change

			CSD2	2.5 ↓	10 ↓

The relative abundance of all the pre-miRNAs is depicted in the form of histogram (Figure 5a, right panel). The precursors of miR162, miR172, miR395, miR397 and miR399 were induced to more than five folds in ToLCNDV infected leaves, while those of miR159, miR160, miR167 and miR319 showed almost 2-3 times increase in ToLCNDV infected leaves (Table 3). Drastic down-regulation (~10 folds) was observed in the expression of pre- miR166a and pre-miR166b (targeting HD-ZIP transcription factors) while pre-miR171 was found to be reduced by two folds (Figure 5a). It can be noted that pre-miRNA sequences of miR166a and miR166b are very different from each other. Interestingly, with flower tissues, no significant changes were observed for miR160 and miR172 precursor levels (Figure 5b) during ToLCNDV infection. Few pre-miRNA transcripts *viz*., miR395, miR397 and miR399 were up-regulated both in leaves and flowers (Figure 5a and 5b) while pre-miR159 and pre-miR171 were down-regulated by 2 folds in flower tissues (Figure 5b). Pre-miR166a and pre-miR166b exhibited induction in infected flowers while their levels in infected leaves were significantly low compared to healthy leaf tissues (Figure 5a and 5b). These results further support that the virus infection induced specific changes in the leaf tissues and such changes might allow the viruses to establish the leaf specific disease phenotype.

### Semi-quantitative reverse transcription-PCR of the miRNA targets

**Figure 6:**
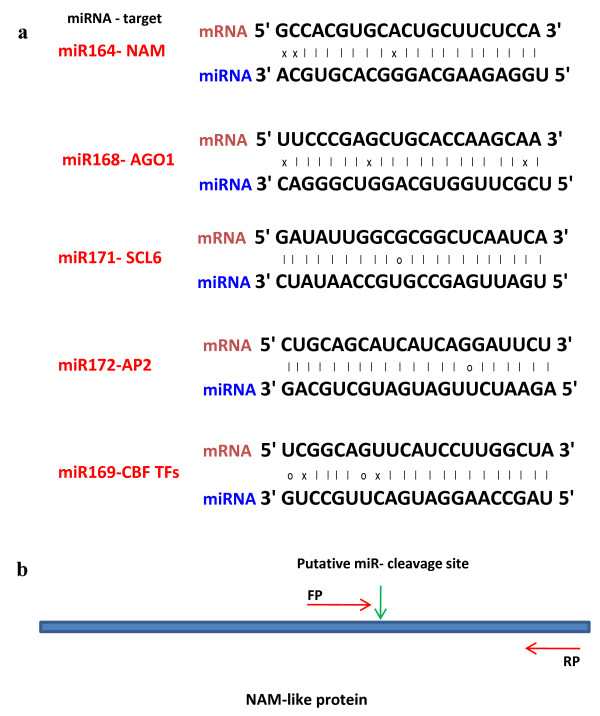
***In silico *prediction of miRNA targets using tomato EST database**. **a**, The Sequence alignment between miRNAs and their respective targets used in the expression analysis. The symbols (×) refer to mismatch while (○) refers to wobble base pairing. **b**, The primers designed for RT-PCR based expression analysis flanked the putative miRNA cleavage site, in case of miR164 targeted NAM-like gene.

Plant miRNAs show tight complementarity to their targets that leads to the target cleavage and degradation. Thus, the miRNA and its target essentially show mutually antagonistic expression levels. To test whether such correlation exists between the deregulated miRNA levels and their corresponding targets, we performed semi-quantitative reverse transcription-PCR analysis of the few miRNA (including both up- and down-regulated) - targets. The tomato ESTs exhibiting strong sequence complementarity with respective miRNAs (Figure 6a) were selected for further analysis. These ESTs share sequence homology in other plant species and are also targeted by same miRNAs of the corresponding plants. Total RNA, isolated from leaves and flowers tissues, was reverse transcribed with oligo-dT and amplified using gene specific primers (see Additional file 2; Table S 2) designed from the target sequences as obtained from tomato EST database. Figure 6a shows sequence alignment for few of the targets and their respective miRNA. The RT-PCR primers flanked the putative cleavage site in case of some target genes e.g, NAM-like protein which is targeted by miR164 (Figure 6b).

**Figure 7:**
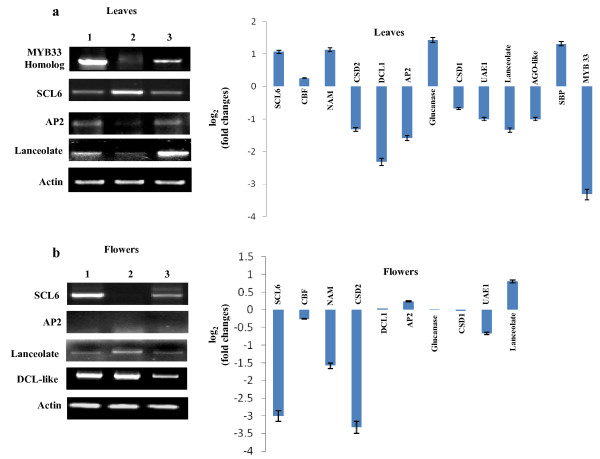
**Semi-quantitative Reverse transcription Polymerase chain reaction (RT-PCR) of various miRNA-targeted genes in (a) leaves and (b) flower tissues of different plants: healthy Pusa Ruby (lane 1), ToLCNDV (2A+2B) agroinfected Pusa Ruby (lane 2) and LA1777 (lane 3)**. The RT-PCR data of only few targets are displayed on the left of each panel and the fold changes of the individual targets as a result of virus infection were calculated (as mentioned above). The calculated changes have been shown on the right of each panel for a large number of targets. The band intensity of the targets was normalized with respect to actin amplification. The experiments were conducted twice.

Targets of the miRNAs with induced levels in response to ToLCNDV infection were generally down-regulated in leaves as observed from RT-PCR analyses (Figure 7). These results show that DCL1, CSD1, CSD2, AGO1, MYB33 homolog, lanceolate and UAE1 transcript levels were reduced significantly (2-10 fold) in ToLCNDV agroinfected leaves compared to their counterparts of the healthy tissues (Table 3 and Figure 7a). This suggests that these deregulated miRNAs were functional to down regulate the expression of their respective targets in leaves. However, endo 1-4- beta glucanase (referred to as glucanase in the Figure 7a), a target of miR396, did not show expected expression. The miR396 was induced two-folds in the ToLCNDV infected leaves but the target gene (endo 1-4- beta glucanase) also exhibited upregulation by 2.5 folds. However, UAE1, another predicted target of miR396 was down-regulated in ToLCNDV infected leaves compared to healthy control. It seems that some of the ToLCNDV induced miRs (such as miR396) might not be functional with regard to the gene-silencing activity. Alternatively, miR396 does not target endo1-4-beta glucanase gene in tomato. When the expression levels of the targets were checked among corresponding flower tissues, interestingly, we did not notice any significant changes in most of the genes studied. For example, targets like DCL1, AP2, endo 1-4- beta glucanase and CSD1 were expressed to almost similar extent, at least, in Healthy Pusa Ruby and ToLCNDV infected flowers. From Figure 7b, we noticed the down-regulation of other targets studied in flower tissues, including lanceolate (2 fold), CSD2 (10 fold) and UAE1 (1.5 fold). Expression of few genes (MYB33, SBP-like and AGO-like) was not studied in the flower tissues.

Besides, we also carried out the target expression analysis of miRNAs that were down-regulated following ToLCNDV infection. We chose SCL6- like TF, NAM-like TF and CBF TF that are targets of miR171, miR164 and miR169, respectively. All of these targets showed expected increase in the expression levels and were up-regulated by 1.3 to 2 folds in ToLCNDV infected leaves (Table 3 and Figure 7a). The corresponding flower tissues showed marked reductions in the levels of SCL6 (10 folds) and NAM (3 folds) while CBF TF expression was not altered in ToLCNDV infection (Figure 7b). On the basis of target expression analysis, we grouped the genes of the present study in three different sets *viz*., set A that includes the targets down-regulated by almost 5 folds (DCL1, Apetala2 and MYB33), set B with genes showing two-fold reduction (CSD1, CSD2, UAE 1and AGO1) while set C includes targets that were up-regulated (SBP, SCL6 and NAM) in ToLCNDV infected leaves. MiR169 (slightly down-regulated in ToLCNDV infected leaves) targeted CBF transcript did not show significant changes in the expression level. The expression pattern of the miRNA targets observed in RT-PCR analysis was further confirmed by performing northern hybridization for few genes *viz*., MYB33 homolog, Apetala 2, CSD1, endo 1-4 beta glucanase and DCL1. We observed that MYB33 homolog, Apetala 2, CSD1 and DCL1 transcripts were significantly reduced in ToLCNDV agroinfected leaves compared to healthy Pusa Ruby and LA1777 leaves (data not shown). The endo 1-4 beta glucanase transcripts showed similar expression as was observed in RT-PCR results (data not shown). Thus, RT-PCR results support our northern blot data.

### Conserved and dynamic miRNA expression during leaf curl disease

**Figure 8:**
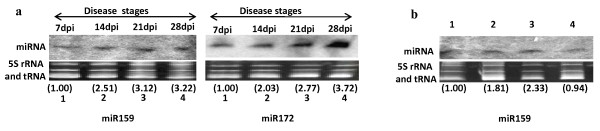
**Northern analysis of miR159 and miR172 at different days post inoculation (dpi) of ToLCNDV construct. **a, Leaf samples obtained at 7, 14, 21, and 28 dpi of ToLCNDV agroinoculated tomato cv Pusa Ruby plants. Levels of miR159 and miR172 increase significantly as the ToLCNDV disease symptoms progress. The expression levels were normalized with respect to the ethidium bromide stained RNA bands. The calculated normalized levels are shown in parenthesis. **b**, The levels of miR159 were analyzed and found to induce in tomato ToLCNDV (2A+2B) infected cv JK Asha and chilli plants showing altered leaf morphology. **1**: Healthy chilli leaves; **2**: ToLCNDV (2A+2B) infected chilli leaves; **3**: ToLCNDV (2A+2B) infected cv JK Asha leaves; **4**: Healthy cv JK Asha. The EtBr stained gels serve as loading control.

To show further that some of the deregulated miRNAs are tightly associated with ToLCNDV infection, we analyzed their expression levels with increasing dpi of ToLCNDV (2A+2B) agroinoculation. Based on the severity of the symptoms, we classified ToLCNDV disease in three stages *viz*., development of early disease (stage A, 7 dpi), late (stage B, 14-21 dpi) and late severe disease (stage C, 28 dpi). We obtained two different samples representing stage B and were named as B1 (14dpi) and B2 (21 dpi). At B2 stage, leaf-curling as well as yellow mosaic symptoms were visible whereas at the B1 stage, there was only leaf curling. The leaves of ToLCNDV agroinfected plants were checked for the levels of miR159 and miR172 at different dpi since these miRNAs showed induced expression levels in microarray results. Northern hybridization results clearly showed that miR159 and miR172 expression levels were induced with increasing dpi of ToLCNDV infection and this accumulation was directly linked to the disease severity (Figure 8a). Leaves obtained at 28dpi, showed maximum expression of both, miR159 (~3.2 folds) and miR172 (~3.7 folds). To further substantiate our results, we checked expression levels of miR159 in ToLCNDV agroinfected tomato cv JK Asha and chilli (*Capsicum annum*) that show leaf curl symptom following infection. It is evident from our results that miR159 levels were upregulated by 2.3 folds in ToLCNDV (2A+2B) agroinfected cv JK Asha leaves compared to healthy leaves (Figure 8b; lanes 3 and 4). Moreover, we observed 1.8 folds increase in the expression of miR159 in agroinfected and diseased chilli leaves compared to the uninfected counterpart (Figure 8b; lanes 1and 2). These observations lend strong support to our previous observation that miR159, miR319 and miR172 levels increase with the disease progression [33].

Together, these results strongly suggest that certain miRNAs (e.g., miR159/319 and miR172) could be associated with the ToLCNDV infection as well as pathogenesis.

## Discussion

The involvement of miRNAs in diverse abiotic responses (salt, temperature, drought and nutritional starvation) has been demonstrated by various research groups [23,24,34]. Recent studies have also confirmed their roles in conferring immunity against bacterial responses [35]. Although siRNA-mediated viral defense responses are well studied in plants, the roles of host miRNAs in plant viral immunity/sensitivity have not been well investigated. The miRNA profiling is a good indicator of many diseases, especially cancers, where strategies to cure rely on the early disease detection [36]. There is growing evidence that certain cancerous tissues exhibit deregulated levels of miRNAs, thus supporting the notion that these molecules are promising therapeutic agents or drug targets [37,38]. However, the plant miRNAs as biomarkers of disease are at the stage of exploration. Here, we examined the possibility that miRNAs can be used as diagnostic markers in response to a leaf curl disease caused by ToLCNDV agroinfection in tomato plants. These miRNA biomarkers can eventually be manipulated developing antiviral strategies.

In our present study, we have identified a set of miRNAs, the levels of which were differentially altered under ToLCNDV (2A+2B) infection in tomato leaves. We speculate that both virus and host utilize miRNAs as efficient weapons to fight against each other. A major proportion of the genes targeted by these miRNAs are reported to play crucial role in various defense responses highlighting their role in viral defense mechanism. For instance, the genes targeted by miR398, miR399, miR162 and miR168 are, either directly or indirectly, responsible for responding to adverse stress stimuli. Interestingly, we found upregulation of miR168 and miR162 in our microarray data. These miRNAs target DCL1 and AGO1 proteins respectively, that are primarily involved in regulating global miRNA flux and their functionality can profoundly affect the overall miRNA levels [39,40]. Although DCL1 and AGO1 are demonstrated to participate predominantly in miRNA biogenesis, recent studies have extended their roles in the generation of certain siRNAs. For instance, DCL1 is shown to be required during ta-siRNAs and lsiRNA/nat-siRNAs production and these are necessary in both abiotic and biotic stress responses [41,42]. The miR398 targets Copper superoxide dismutases (CSD1 and CSD2) that are known to control abiotic stress response [43] and we also observed increase in the levels of miR398 following ToLCNDV infection. Thus a cross-talk between the pathways of abiotic and biotic stress-responses might be a reality. Similarly, most of the miRNAs (miR395, miR396 and miR399) known to be involved in abiotic stress conditions showed enhanced expression under ToLCNDV infection and the levels of the corresponding targets were reduced. These target genes are required to overcome oxidative and nutrient stress responses and their repression could be beneficial for establishment of viral infection and disease expression. Till date, only few miRNAs have been identified to regulate leaf development *viz*., miR165/166, miR159/319, miR164 and miR160. We also observe modest changes in the levels of either precursor or mature miRNAs. In addition, our data reflect that many other miRNAs also exhibit altered expression in response to viral infection, suggesting their probable role in basal defense activity and leaf morphogenesis.

Microarray and northern hybridization results show that most of the deregulated miRNAs were induced and only few (miR160, miR164, miR169, miR171 and miR391) were down-regulated following ToLCNDV infection. AC4 of *African Cassava mosaic virus *(ACMV) has been demonstrated to destabilize single stranded miRNAs by interacting directly with them and thus AC4 over-expressing transgenic plants showed reduced accumulation of miRNAs [27]. It is possible that, similar to ACMV AC4, ToLCNDV AC4 might act to destabilize miRNAs which explains the reduction in the levels of certain miRNAs. However, as ToLCNDV infected leaves predominantly show induction in the expression of miRNAs, it likely appears that there exist some other mechanisms to achieve this deregulation both at the miRNA and pre-miRNA stages. ToLCNDV encoded suppressors, *viz*., AC2 and the pre-coat protein (AV2), might also increase the level of miRs and all of these three VSRs (AC2, AC4 and AV2) might behave differently [44].

The RT-PCR analysis of pre-miRNAs, in most of the cases, reveals that the expression of pre-miRs was modulated analogous to their respective mature miRNAs. This suggests that ToLCNDV infection might lead to altered transcription of certain miRNA genes. Since AC2 and CP are known to localize into host nucleus, it is plausible to assume that these VSRs might interact with host transcription factors (TFs) and can modulate transcription of several downstream genes including those of the miRNAs [29,45]. We speculate that ToLCNDV infection leads to transcriptional activation/repression of certain miRNA genes and at the same time certain ToLCNDV encoded VSRs can bind to and stabilize/destabilize both, pre- and/or mature miRNAs.

ToLCNDV spreads systemically, but the virus is abundant in leaves and rarely present in flowers (Figure 1b). Similarly, the virus induced changes in the transcriptome (including miRNA genes) between the leaves and the flower tissues were observed to be dramatically different. Expression studies of pre-miRNA and miRNA targets in flowers, reveals that most of these transcripts did not show levels similar to that observed in leaf tissues. Nonetheless, the flowers of ToLCNDV agroinfected plants exhibit the disease symptoms (Figure 1a). Further, since leaf tissues are the primary site for viral entry, they suffer drastic alteration in the transcriptome following ToLCNDV infection. Therefore, it is not surprising that the deregulation of ToLCNDV induced transcript remain mostly localized in the leaf tissues.

The expression analysis of miR159 and miR172 at different dpi of ToLCNDV (2A+2B) infected Pusa Ruby leaves clearly shows induction of these miRNA during disease progression (Figure 8a). These results suggest that miR159 and miR172 could be used as potential indicator of ToLCNDV infection. Since the miR159 targets (MYB TFs) are well established factors determining leaf structure, the observed leaf deformation in ToLCNDV infected tomato plants could be due to the altered levels of miR159. Another viral induced miRNA *viz*., miR319 targets TCP TF family member, including TCP4. TCP4 is a well known suppressor of growth in Arabidopsis [46]. The accumulation of miR319 in ToLCNDV infected leaves will bring down the expression of tomato TCP4 homologs. This consequently would lead to uncontrolled cell growth and that might reflect in the form of leaf deformation. Thus, the altered expression of miR159/319 could serve as a prospective indicator of leaf curl disease.

Our microarray data revealed that the levels of miR396 were induced (~2 folds) in ToLCNDV infected leaves. MiR396 targets Growth-Regulating Factor (GRFs) transcription factors that are involved in cell division. Moreover, either miR396 over-expression or *GRF *mutation leads to reduced leaf size in Arabidopsis [47,48] and it is noteworthy that the leaves of ToLCNDV agroinfected plants were also of small size compared to those of the uninfected plants. Further, studies by Rodriguez et al. [2010; [49]] demonstrated that miR396 levels were increased in Arabidopsis *soj8 *mutants (miR319-resistant TCP4 lines). Thus, it appears that leaf curl phenotype observed during ToLCNDV agroinfection could be a manifestation of high levels of miR319 and miR396, where both might act synergistically leading to a pronounced leaf curling. Significantly, we also compared the results of agroinfection with the same of field-infection. The induction of various miRNAs and the levels of disease progression were almost similar in both cases. Thus, the conclusions of the additional field level data reinforced the notions derived from the agroinoculation studies.

All these data together indicate that the defense responses are mediated by induction and repression of large array of genes that includes the miRNA transcripts. Although some of these altered transcripts could help host to defend against diverse pathogens and abiotic stresses, viruses can also utilize this disturbed transcriptome for their own benefit i.e., to make suitable environment for their survival.

## Conclusions

The ToLCNDV (2A+2B) agroinfection significantly changes the expression pattern of various miRNAs in tomato leaves. This mis-expression of miRNAs could be a manifestation of altered transcription of corresponding pre-miRs. Moreover, the infected flowers tissues exhibit different expression pattern both of miRNA and corresponding pre-miRNA, indicating the leaf specific effects of virus infection. The targets of these miRs were also altered in the infected tomato leaves and thus, leading to deformed leaf morphology. Few of the deregulated miRNAs were tested in several infection stages as well as in different ToLCNDV (2A+2B) agroinfected plants (tomato and chilli) and were found to be linked with the disease progression. Overall, our study provides the significance of host miRNAs in response to viral infection and that they could likely contribute to viral pathogenesis.

## Methods

### Plant materials used in the study

Tomato cv Pusa Ruby seeds were obtained from IARI, New Delhi while tomato accession LA1777 and cv 15 SB SB were collected from Dr. Manoj Prasad, NIPGR, New Delhi. Seeds were soaked in water and were kept in germinating sheets for germination at 30°C in an incubator. At the seedling stage these were transferred to vermiculite for further growth in green house (28°C, 14 hours light and 10 hours dark, 70% humidity) to be used as healthy controls for experiments. For natural ToLCNDV infection, tomato Pusa Ruby was shifted to the net house and the net was kept open for 5 hours every day for whiteflies to feed. The tissue samples were collected (mainly during the months from June to October period) from plants showing curled leaves and were checked for the presence of virus by PCR or rolling circle amplification (RCA) before proceeding for further experiments.

### Agroinfiltration of tomato leaves

Constructs containing dimer of ToLCNDV-A (Accession DQ629101.1) and ToLCNDV-B (Accession DQ169057) genomes were used for agroinfiltration experiments. *Agrobacterium tumefaciens *LBA4404 was transformed with these constructs. These transformed cells were grown overnight in LB media and the cells were harvested. The cells were resuspended in MES buffer (100mM MES and 500mM MgCl_2_) and the population of DNA-A and DNA-B containing cells were mixed in equal concentrations with final OD between 0.4-0.6. The mixed culture (~1 ml) was infiltrated into tomato leaves abaxially using syringe.

### Rolling Circle Amplification (RCA) for the detection of viral replicon

Genomic DNA was isolated from the leaves of agroinfiltrated and mock plants using CTAB method. Approximately 100 ng of gDNA from each sample was used for RCR reaction, as per the manufacturer's instructions (TempliPhi™, GE Healthcare). The amplified products were digested with EcoRI enzyme (New England Biolabs) to release the monomers from the concatamers of ~2.5 kb. The digested products were run on 1% agarose gel and the amplified replicon was visualized by Et-Br staining. The DNA was sequenced and was found to be ~2610 bp long as the ToLCNDV-A (Accession DQ629101.1) had three EcoR1 sites.

### MicroRNA Array Profiling

Total RNA isolated from the leaf samples obtained from tomato cv Pusa Ruby Healthy, ToLCNDV (2A+2B) agroinfiltrated Pusa Ruby (21 dpi), healthy LA1777, ToLCNDV (2A+2B) agroinfiltrated tomato cv 15 SB SB and mock infiltrated tomato cv 15 SB SB were custom analyzed for global miRNA expression by EXIQON, Denmark. The protocol for the methodology (as provided by the company) employed is briefly described here. Approximately 500 ng total RNA from sample and reference were labeled with Hy3™ and Hy5™ fluorescent label, respectively. The Hy3™-labeled samples and a Hy5™-labeled reference RNA sample were mixed pair-wise and hybridized to the miRCURY™ LNA array version 11.0 (Exiqon, Denmark), which contains capture probes targeting miRNAs deposited on Sanger's database (Release 12). The hybridization was performed according to the miRCURY™ LNA array manual using a Tecan HS4800 hybridization station (Tecan, Austria). The miRCURY™ LNA array microarray slides were scanned using the Agilent G2565BA Microarray Scanner System (Agilent Technologies, Inc., USA) and the image analysis was carried out using the ImaGene 8.0 software (BioDiscovery, Inc., USA). The quantified signals were background corrected (Normexp with offset value 10) and normalized using the global Lowess (LOcally WEighted Scatterplot Smoothing) regression algorithm. The miRNAs exhibiting statistically significant expression differences were represented on the heat map. The data from the heat map were used to derive values of difference in expression (Δ Expresssion) of any particular miR across various samples. From the data-sets, histograms of deregulation of miRs were plotted (see Additional file 1, Fig. S 2).

### Small RNA gel blots analysis

Total RNA was isolated from healthy Pusa Ruby, ToLCNDV (2A+2B) agroinfected Pusa Ruby and LA1777 leaves using RNeasy plant mini kit (Qiagen). Low molecular weight RNA was isolated from total RNA using Polyethyleneglycol (PEG) and NaCl precipitation. Approximately 150 micrograms of total RNA was incubated with PEG (final concentration 5%) and NaCl (final concentration 1M) in ice for 30 minutes, centrifuged 13000 rpm for 15 minutes and the pellet was resuspended in appropriate volume of DEPC- treated water. Ten micrograms of enriched RNA was resolved on a 15% denaturing polyacrylamide gel, and transferred electrophoretically to Hybond-N+ membranes (GE healthcare) and was UV cross-linked. For the preparation of probes, DNA oligonucleotides complementary to the miRNA sequences (listed below) were end labeled with γ-^32^P-ATP (6000 Ci/mmol; Perkin Elmer Life Scineces, USA) using T4 polynucleotide kinase (New England Biolabs). The probes were purified using G25 columns (GE healthcare). Membranes were incubated with pre-hybridization buffer for at least 3 hours, followed by overnight hybridization with labeled probe at 40°C. Blots were washed twice (for 20 minutes) with 1× SSC, 0.2% SDS at 50°C. The membranes were air dried and exposed to phosphorscreen and images were acquired by scanning the films with a Typhoon (GE healthcare). The DNA oligos used as probes for northern analysis are given below:

miR159: 5' - TAGAGCTCCCTTCAATCCAAA- 3';

miR164: 5' - TGCACGTGCCCTGCTTCTCCA- 3';

miR171: 5' - AGATGATATTGGCACGGCTCA- 3';

miR172: 5' - ATGCAGCATCATCAAGATTCT -3';

miR319: 5' - CTTGGACTGAAGGGAGCTCC-3';

### miRNA precursor expression analysis by semi-quantitative Reverse transcription-PCR

Total RNA from healthy Pusa Ruby, ToLCNDV (2A+2B) agroinfected Pusa Ruby and LA1777 leaves and flowers was prepared using an RNeasy plant mini kit (Qiagen). First strand cDNA was synthesized from total RNA (2 μg) with oligo-dT as reverse primer using SuperScript III (Invitrogen, Carlsbad, CA, USA). This cDNA was directly used to perform semi-quantitative reverse transcription (RT)-PCR using gene specific primers (see Additional file 2; Table S 1) designed from pre-miR sequences procured from miRBase (release 13). For normalization of the transcript expression levels, actin served as an internal control and the reaction was carried out at varying cycles for proper quantification of transcripts. For actin, the RT-PCR was carried out at low cycles to avoid saturation of band intensity. The RT reaction mix without reverse transcriptase served as a negative control. After the linear phase of DNA amplification (varying from 25-30 cycles), the PCR products were resolved by electrophoresis on a 2.0% agarose gel alongwith 100 bp DNA ladder (Fermentas) as molecular weight marker.

### Expression analysis of miR-targeted transcripts by semi-quantitative Reverse transcription-PCR

Total RNA from tomato healthy Pusa Ruby, ToLCNDV (2A+2B) agroinfected Pusa Ruby and LA1777 leaves and flowers was prepared using an RNeasy plant mini kit (Qiagen). Two micrograms of total RNA was used for oligo-dT-primed first-strand cDNA synthesis with SuperScript III ™, (Invitrogen). PCR amplification (26 cycles) of cDNA was performed using primers (see Additional file 2; Table S 2) to selectively amplify genes. Actin gene was also amplified and used as an internal control.

## Competing interests

The authors declare that they have no competing interests.

## Authors' contributions

ARN carried out all the experiments and wrote the manuscript. QMRH helped in drafting the manuscript. SKM conceived of the study, and participated in its design and wrote the manuscript in its final form. All authors read and approved the final manuscript.

## Supplementary Material

Additional file 1 **Supplemental Figures**. The file contains three figures as supporting data and has been mentioned in the text. The legends to these supplemental figures are provided below. **Figure S 1**. **Detection of viral amplicon in the agro-inoculated tomato plants using Rolling circle amplification (RCA)**. The agarose gel shows the presence of viral DNA in agro-inoculated plants, employing the RCA technique on the genomic DNA isolated from tomato leaves. Approximately 2.5 kb band correspond to ToLCNDV A and B genome. It may be noted that from the restriction analysis of DNA A and DNA B genomes release product of similar size (~2.5 kb). **Figure S 2. Histograms of the expression of miRNAs as derived from the heat map**. The quantitative expression values of miRNAs that are either (**A**) upregulated or (**B**) downregulated in response to ToLCNDV (2A+2B) agroinfection are shown and compared with other samples. The y-axis represents the log_2 _(Hy3/Hy5) ratios. **Figure S 3. Northern blot analysis of miRNAs down-regulated in response to ToLCNDV (2A+2B) infection**. The expression levels of miR164 and miR171 were markedly reduced in ToLCNDV (2A+2B) infected leaf samples. The normalization of expression of individual miRNAs was performed with respect to EtBr stained RNA gels and further quantified. The value corresponding to healthy sample is taken as 1 while the values presented for other samples are with respect to healthy controls. The quantified values for each miRNA are shown in parenthesis below each sample lane. EtBr stained gels serve as loading control. **1**: Pusa Ruby healthy; **2**: ToLCNDV (2A+2B) agroinfected Pusa Ruby; **3**: LA1777 healthy. Click here for file

Additional file 2 **Supplemental Tables**. There are two supplemental tables which enlists the primers used in this study. **Table S 1**: The list of primers used to amplify pre-miRNAs from the tomato cDNA library. **Table S 2**: The list of primers used to amplify miRNA targets from the tomato cDNA library. Click here for file
