# Supplementary material for: MicroRNA profiling of tomato leaf curl new delhi virus (tolcndv) infected tomato leaves indicates that deregulation of mir159/319 and mir172 might be linked with leaf curl disease
Source: Virol J. 2010 Oct 25;7:281. doi: 10.1186/1743-422X-7-281 (PMC2972279; doi:10.1186/1743-422X-7-281)
Supplement: Additional file 1 — Supplemental Figures. The file contains three figures as supporting data and has been mentioned in the text. The legends to these supplemental figures are provided below. Figure S 1. Detection of viral amplicon in the agro-inoculated tomato plants using Rolling circle amplification (RCA). The agarose gel shows the presence of viral DNA in agro-inoculated plants, employing the RCA technique on the genomic DNA isolated from tomato leaves. Approximately 2.5 kb band correspond to ToLCNDV A and B genome. It may be noted that from the restriction analysis of DNA A and DNA B genomes release product of similar size (~2.5 kb). Figure S 2. Histograms of the expression of miRNAs as derived from the heat map. The quantitative expression values of miRNAs that are either (A) upregulated or (B) downregulated in response to ToLCNDV (2A+2B) agroinfection are shown and compared with other samples. The y-axis represents the log2 (Hy3/Hy5) ratios. Figure S 3. Northern blot analysis of miRNAs down-regulated in response to ToLCNDV (2A+2B) infection. The expression levels of miR164 and miR171 were markedly reduced in ToLCNDV (2A+2B) infected leaf samples. The normalization of expression of individual miRNAs was performed with respect to EtBr stained RNA gels and further quantified. The value corresponding to healthy sample is taken as 1 while the values presented for other samples are with respect to healthy controls. The quantified values for each miRNA are shown in parenthesis below each sample lane. EtBr stained gels serve as loading control. 1: Pusa Ruby healthy; 2: ToLCNDV (2A+2B) agroinfected Pusa Ruby; 3: LA1777 healthy. [file 1743-422X-7-281-S1.PPT]

## Slide 1
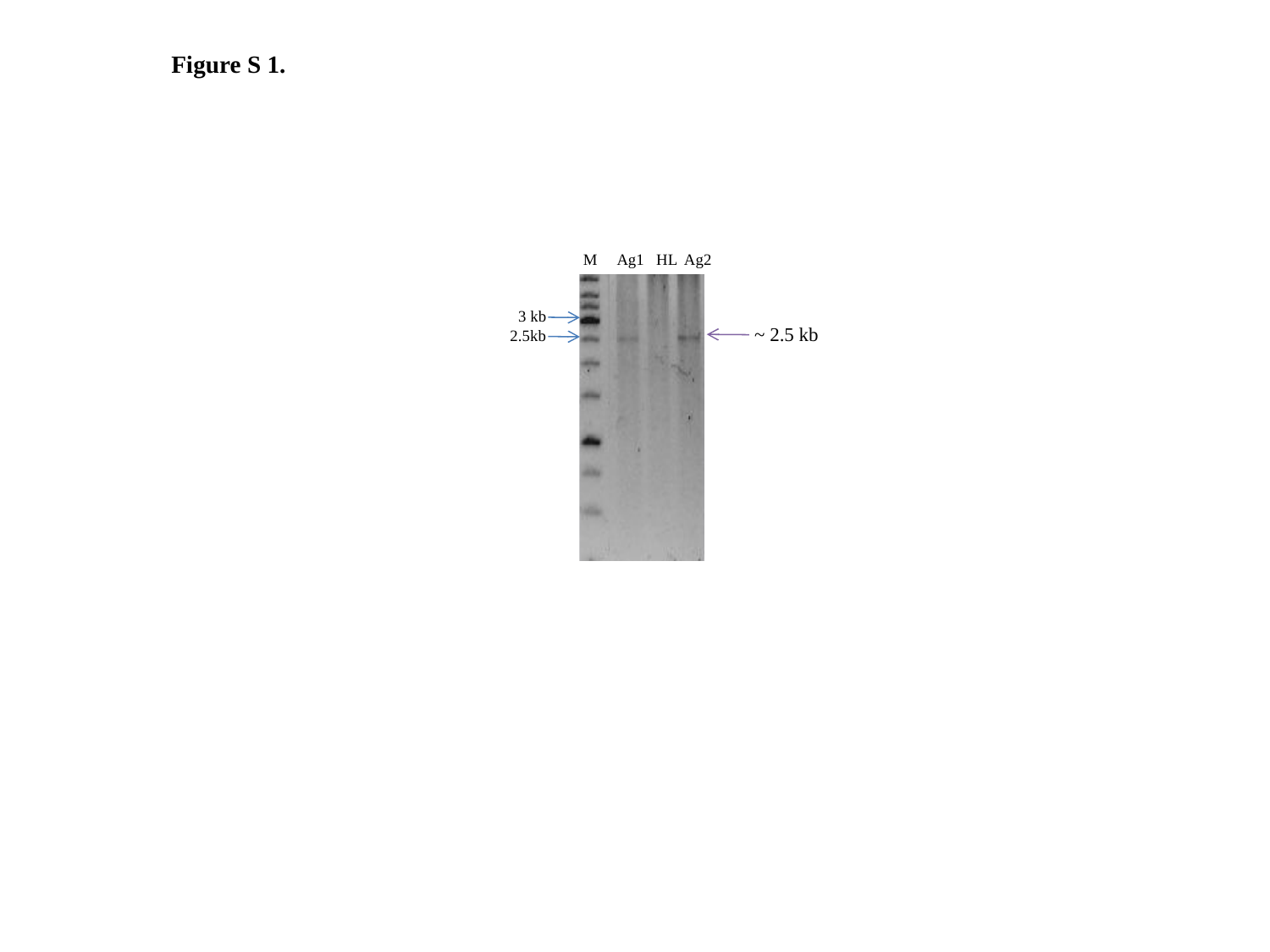

Figure S 1.
M Ag1 HL Ag2
~ 2.5 kb
3 kb
2.5kb

## Slide 2
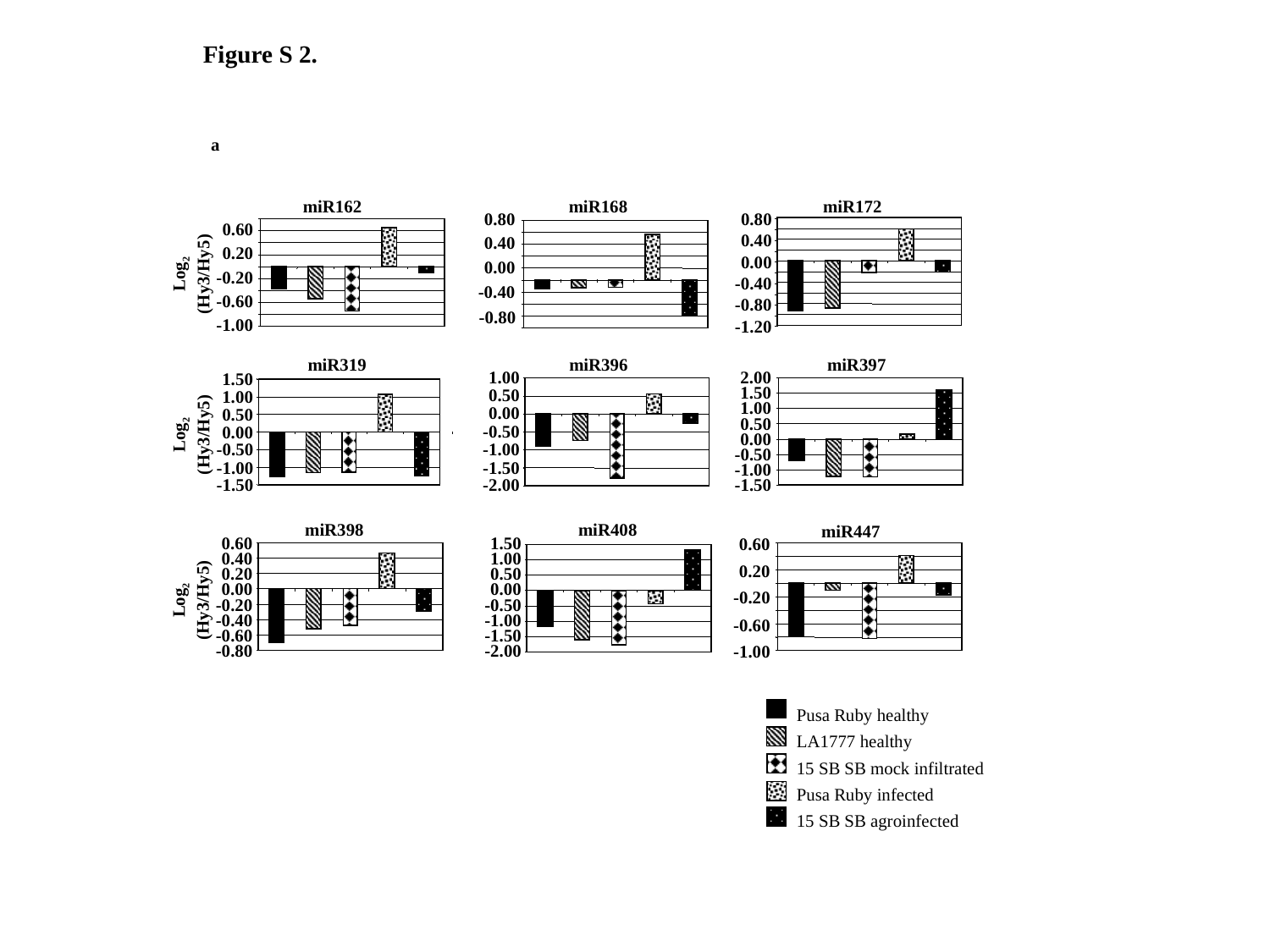

Figure S 2.
a
miR162
miR168
miR172
0.80
0.40
0.00
-0.40
-0.80
-1.20
0.80
0.40
0.00
-0.40
-0.80
0.60
0.20
-0.20
-0.60
-1.00
Log2
(Hy3/Hy5)
miR319
1.50
1.00
0.50
0.00
-0.50
-1.00
-1.50
miR396
1.00
0.50
0.00
-0.50
-1.00
-1.50
-2.00
miR397
2.00
1.50
1.00
0.50
0.00
-0.50
-1.00
-1.50
Log2
(Hy3/Hy5)
miR398
0.60
0.40
0.20
0.00
-0.20
-0.40
-0.60
-0.80
miR408
1.50
1.00
0.50
0.00
-0.50
-1.00
-1.50
-2.00
miR447
0.60
0.20
-0.20
-0.60
-1.00
Log2
(Hy3/Hy5)
Pusa Ruby healthy
LA1777 healthy
15 SB SB mock infiltrated
Pusa Ruby infected
15 SB SB agroinfected

## Slide 3
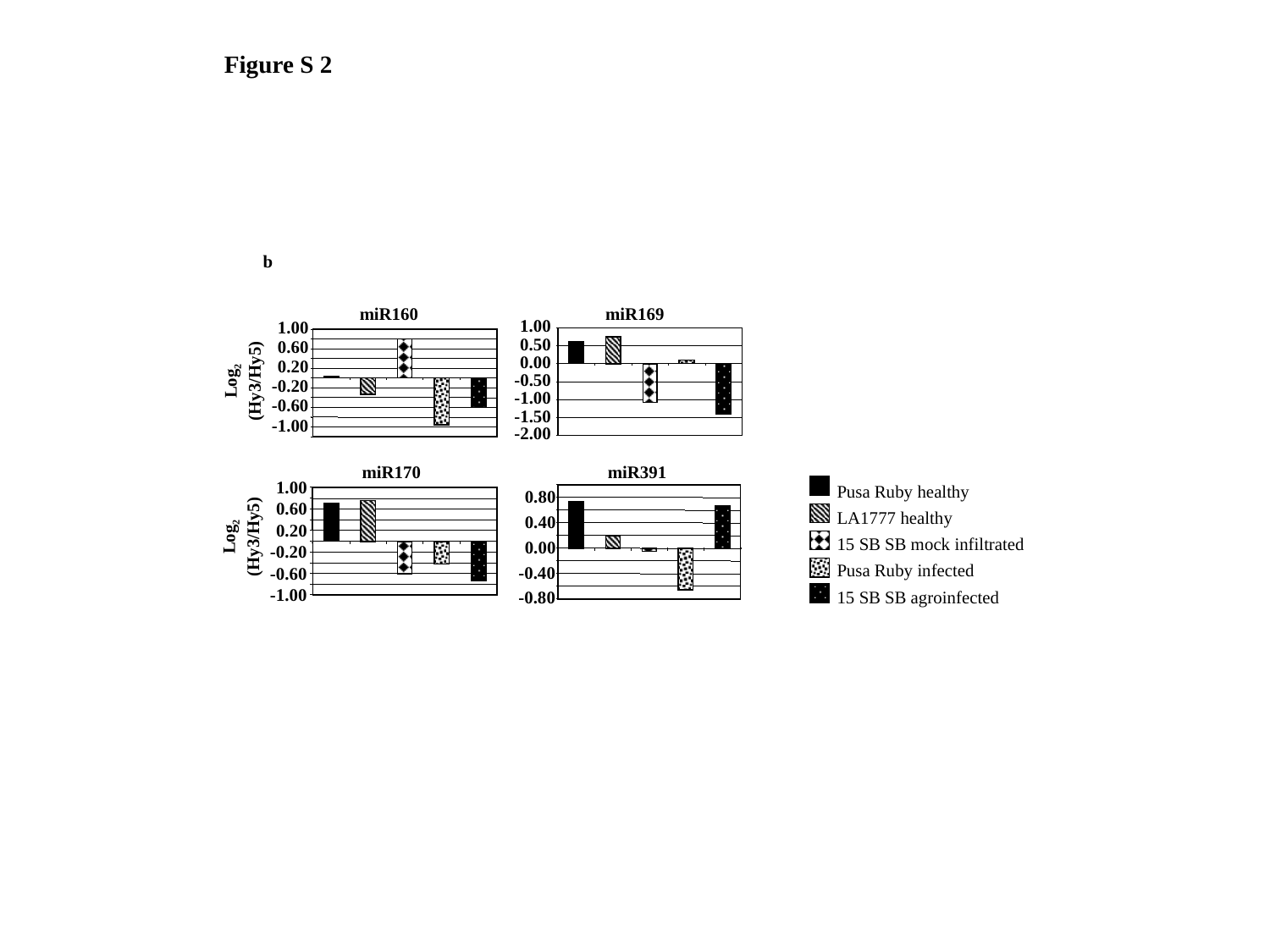

Figure S 2
b
miR160
1.00
0.60
0.20
-0.20
-0.60
-1.00
miR169
1.00
0.50
0.00
-0.50
-1.00
-1.50
-2.00
Log2
(Hy3/Hy5)
miR170
miR391
0.80
0.40
0.00
-0.40
-0.80
Pusa Ruby healthy
LA1777 healthy
15 SB SB mock infiltrated
Pusa Ruby infected
15 SB SB agroinfected
1.00
0.60
0.20
-0.20
-0.60
-1.00
Log2
(Hy3/Hy5)

## Slide 4
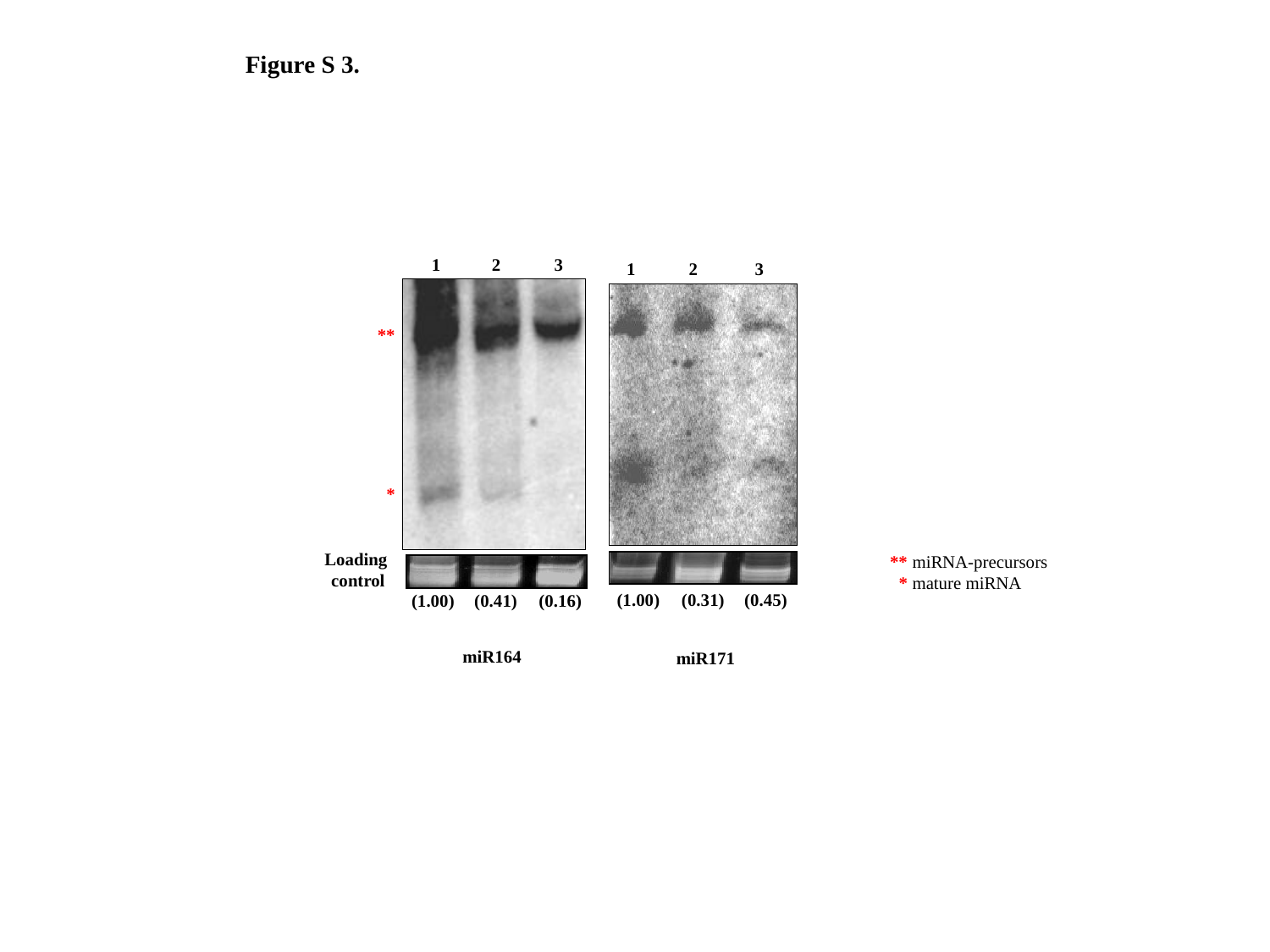

Figure S 3.
1
2
3
(1.00)
(0.41)
(0.16)
1
2
3
(1.00)
(0.31)
(0.45)
miR164
miR171
**
*
Loading
control
** miRNA-precursors
 * mature miRNA
