# Supplementary material for: MicroRNA profiling of tomato leaf curl new delhi virus (tolcndv) infected tomato leaves indicates that deregulation of mir159/319 and mir172 might be linked with leaf curl disease
Source: Virol J. 2010 Oct 25;7:281. doi: 10.1186/1743-422X-7-281 (PMC2972279; doi:10.1186/1743-422X-7-281)
Supplement: Additional file 2 — Supplemental Tables. There are two supplemental tables which enlists the primers used in this study. Table S 1: The list of primers used to amplify pre-miRNAs from the tomato cDNA library. Table S 2: The list of primers used to amplify miRNA targets from the tomato cDNA library. [file 1743-422X-7-281-S2.DOC]

**Table S 1.** The list of primers used to amplify pre-miRNAs from the tomato cDNA library**.**

| Pre-miR 171 Forward | 5’-CAGTAACTTTGATATTGGCC-3’ |
| --- | --- |
| Pre-miR 171 Reverse | 5’-CTGAGATATTGGCACGGCTC- 3’ |
| Pre-miR 159 Forward | 5’-TGGAGCTCCTTGAAGTCCAAC-3’ |
| Pre-miR 159 Reverse | 5’-TAGAGCTCCCTTCAATCC-3’ |
| Pre-miR 166a Forward | 5’-GTTGAGGAGAATGTCGTCTGG-3’ |
| Pre-miR 166a Reverse | 5’-GTTGAGGGGAATGAAGCCT-3’ |
| Pre-miR 166b Forward | 5’-CCATAGATCATATGGAGGAA-3’ |
| Pre-miR 166b Reverse | 5’-CCTAGCTAGAGGAGTAACA-3’ |
| Pre-miR 319 Forward | 5’-GTCCACACATGGGGAACGATAG-3’ |
| Pre-miR 319 Reverse | 5’-GGAGCTCCCTTCAGTCCAAG-3’ |
| Pre-miR 397 Forward | 5’-CAGAGTACTTAGAAACATGA-3’ |
| Pre-miR 397 Reverse | 5’-GATCGAGTTTAGCGTTGATG-3’ |
| Pre-miR 399 Forward | 5’-TAGGGCTACACTCTATTGG-3’ |
| Pre-miR 399 Reverse | 5’-TAGGGCAACTCTCCTTTGGC-3’ |
| Pre-miR 395 Forward | 5’-AGGGCTTACTTTGATGTTGG-3’ |
| Pre-miR 395 Reverse | 5’-GCTCTCATGAAGTGGTCAAGGG-3’ |
| Pre-miR167 Forward | 5’-TGAAGCTGCCAGCATGATCTAAAC-3’ |
| Pre- miR167 Reverse | 5’-TAAGGCTGCCACATGATCTG- 3’ |
| Pre- miR160 Forward | 5’-TGCCTGGCTCCCTGTATGCC-3’ |
| miR160 pre Reverse | 5’-GCTTGGCTCCTCATACGCC- 3’ |
| Pre-miR162 Forward | 5’-CTGGAGGCAGCGGTTCATCGA-3’ |
| Pre-miR162 Reverse | 5’-CTGGATGCAGAGGTTTATCG-3’ |
| Pre-miR172 Forward | 5’-ATGTAGCATCATCAAGATTC-3’ |
| Pre- miR172 Reverse | 5’-ATGCAGCATCATCAAGATTC-3’ |

**Table S 2:** The list of primers used to amplify miRNA targets from the tomato cDNA library.

| Coat Protein Forward | 5’-TGTGARGGYCCWTGTAARGTYCA-3’ |
| --- | --- |
| Coat Protein Reverse | 5’-TASARGCATGWGTACANGCCATATAC-3’ |
| Lanceolate Forward | 5’-GACAGTAGAGAAATTGGCCCTG -3’ |
| Lanceolate Reverse | 5’-CACTCCTAATGTAGCTTGTTGAGC-3’ |
| DCL1 Forward | 5’-ACCATGTTGCTTACCATTTGCTG-3’ |
| DCL1 Reverse | 5’-CAGTTATGCTGCATATTATAAGC- 3’ |
| CSD1 Forward | 5’-GAGCTGTTGTTGTTCATGCTGA-3’ |
| CSD1 Reverse | 5’-ACTGCAGGCACTGTAATCTGC-3’ |
| AP2-like Forward | 5’-GCCAACATGATCTTGATCTGA-3’ |
| AP2- like Reverse | 5’-GAATGAAGAATCCTGATGTGC-3’ |
| Glucanase Forward | 5’-GCAATCACTATCCTAAGCATG -3’ |
| Glucanase Reverse | 5’-CATAGTGTGTGTAGATGATACA-3’ |
| UAE1 Forward | 5’-GATGCTGCGGTTATCGATGAACT-3’ |
| UAE1 Reverse | 5’-AGAACATAGAGACTGGAGGATAGTGC-3’ |
| SBP Forward | 5’-CCAGCAATGTAGCAGGTTCCAT-3’ |
| SBP Reverse | 5’-GTGCTCCACACGCTGAAGTTGT- 3’ |
| AGO1-like Forward | 5’-GCGAGGGAATTTGGTATTAAGAT-3’ |
| AGO1-like Reverse | 5’-CAAGGGGAATTCGCCTAGAGATC-3’ |
| SCL6-lke Forward | 5’-TGCTACTCCATGCCAACACCAAC-3’ |
| SCL6-like Reverse | 5’-ACAATCTGCCTATGCCACAAG-3’ |
| CBF Forward | 5’-GGCTCGTGACTGCTTATGGATCA-3’ |
| CBF Reverse | 5’-CATAGCCAAGGATGAACTGCCGAT-3’ |
| CUC2 Forward | 5’-GACAGATTCATCGCCTAGCCA-3’ |
| CUC2 Reverse | 5’-CCTGATCTCATACAATCAC-3’ |
| MYB33 homolog Forward | 5’-GGAGAACACCATGTTGTGAT-3’ |
| MYB33 homolog Reverse | 5’-GATTGATTCAGATGAATCTTCC-3’ |
| CSD2 Forward | 5’-GTCTACAGGTTAGCTTCTGAT-3’ |
| CSD2 Reverse | 5’-GGCAAGTGTGTACATACAAGT-3’ |
| ACTIN Forward | 5’-ATGCCATTCTCCGTCTTGACTTG-3’ |
| ACTIN Reverse | 5’-GAGTTGTATGTAGTCTCGTGGATT-3’ |
